# Home-based balance training using Wii Fit™: a pilot randomised controlled trial with mobile older stroke survivors

**DOI:** 10.1186/s40814-018-0334-0

**Published:** 2018-08-25

**Authors:** André Golla, Tobias Müller, Kai Wohlfarth, Patrick Jahn, Kerstin Mattukat, Wilfried Mau

**Affiliations:** 1 Institute of Rehabilitation Medicine, Medical Faculty, Martin Luther University Halle-Wittenberg, Magdeburger Str. 8, 06112 Halle (Saale), Germany; 2 Department of Neurology, University Hospital Halle (Saale), Ernst-Grube-Str. 40, 06097 Halle (Saale), Germany; 3 Clinic of Neurology, BG Hospital Bergmannstrost Halle (Saale), Merseburger Str. 165, 06112 Halle (Saale), Germany; 4 Nursing Research Unit, University Hospital Halle (Saale), Ernst-Grube-Str. 30, 06120 Halle (Saale), Germany

**Keywords:** Stroke, Balance exercise, Nintendo Wii™, Home rehabilitation, Feasibility

## Abstract

**Background:**

Several studies have reported that using the Wii™ Balance Board can provide added value regarding balance (re-)training in neurological diseases. However, for the large group of mobile older stroke survivors, there is no evidence regarding the feasibility of an unsupervised Wii™ Balance Board training in the home setting. The aim of this study was to investigate the feasibility of a home-based Wii™ balance training for these patients and to identify methodological challenges for randomised controlled trials in the future.

**Methods:**

We conducted a pilot randomised controlled trial with two intervention arms in participants’ homes. Mobile stroke survivors (aged 60 years or above; 12 weeks after discharge from hospital) received a 6-week (once per week) supervised balance training at the study centre, followed by a 6-week (three times per week) unsupervised balance training at home. We used the Nintendo Wii™ Balance Board for one intervention arm and conventional balance exercises for the other intervention arm. Feasibility was assessed by recruiting rates, appropriateness of assessments regarding sensitivity to changes and acceptance of the intervention by the participants.

**Results:**

In two German hospital stroke units, 349 stroke survivors were screened over a period of 6 months, 91 were eligible and 52 were interested. Twelve weeks after discharge, 14 participants agreed and 11 completed the intervention (7 men and 4 women, mean age 74 years). The Berg Balance Scale and Dynamic Gait Index showed ceiling effects already at baseline measure. The participants in both intervention arms evaluated the unsupervised training positively and feasible for self-application. No falls or injuries occurred over the intervention period, while the required scope of the exercises could largely be achieved.

**Conclusions:**

In this pilot study, the recruitment of participants and the chosen assessments were not satisfactory due to selection bias and corresponding ceiling effects. However, the two home-based balance interventions proved feasible for mobile older stroke survivors with low functional limitations.

**Trial registration:**

ClinicalTrials.gov, NCT02251470. Registered 29 September 2014

## Background

Over the past few years, there has been a growing interest in video game-based exercises as a new therapy option in neurological rehabilitation. Besides complex robot-assisted therapy devices [1, 2], low-cost commercially available technologies, such as the game console Nintendo Wii™ (Nintendo Co. Ltd., Minami-ku Kyoto, Japan), have been tested for application in different rehabilitation settings [3]. The Nintendo Wii™ is a motion-controlled game system that can be used with a handheld controller (Wii™ Remote) or a force platform (Wii™ Balance Board) [4]. The technology provides simple visual bio-feedback training for upper or lower limb motor deficits in a playful way. Several studies have reported that using the Wii™ Balance Board can provide added value regarding balance (re-)training in neurological diseases [5–8]. Due to the motivational advantages [9, 10] and the relatively safe training conditions [11–14], the Wii™ Balance Board is considered as an option for supplementary, independent home training. Preliminary (pilot) studies with patients suffering from multiple sclerosis and Parkinson’s disease reported home-based Wii™ Balance Board training to be an effective, well-accepted and safe balance training tool [15–18]. However, for stroke patients, there is no evidence regarding the feasibility of an unsupervised Wii™ Balance Board training at home. Additionally, there is a lack of evidence whether and to what extent Wii™ balance training is more effective than conventional balance exercises [19].

In the context of stroke research, unsupervised intervention trials in a home-based setting imply particular methodological and ethical challenges. These challenges arise from the combination of the special features of this patient group and the unsupervised condition in the home setting. Stroke survivors are often aged 60 years or older [20] and have a high risk of falling (37 to 73% in the first 6 months after hospital discharge [21]). For older adults, exercise adherence after discharge from a physical therapy programme has been shown to be generally poor [22]. Furthermore, the positive experiences with inpatient Nintendo Wii™ balance training [7, 11, 12, 23–26] may not be transferable to the home setting. Therefore, the current study assesses the feasibility of a home-based Nintendo Wii™ balance training for mobile older stroke survivors and collects information about the requirements for the design of a subsequent larger randomised controlled trial.

## Methods

### Study design

**Fig. 1:**
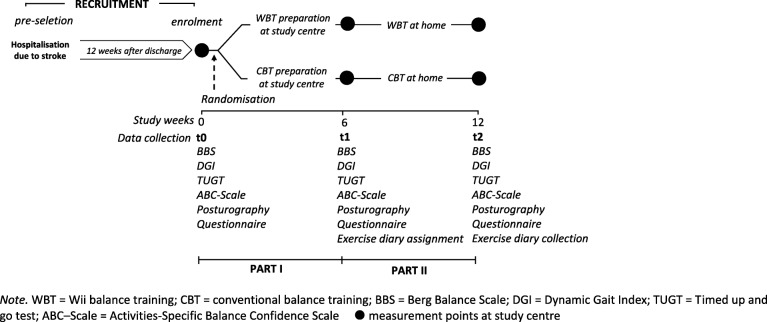
Study design and data collection

This study was a phase II open-label randomised controlled trial (Fig. 1) with two active balance intervention arms and three points in time with data collection: baseline (t0), 6-week follow-up (t1) and 12-week follow-up (t2).

The Ethics Committee of the Medical Faculty of the Martin Luther University Halle-Wittenberg approved the study protocol, and the trial has been registered at ClinicalTrials.gov (NCT02251470). The recommendations of the Medical Research Council framework [27] had been considered for planning the trial while the report of the results was done in accordance with the recommendations for pilot studies [28, 29].

### Recruiting of participants

At two German hospital stroke units in Halle (Saale), Germany, consecutive stroke patients were screened by in-house study nurses for eligibility (August 2014 to February 2015). The study nurses referred to the following inclusion criteria: (I) diagnosis stroke (ICD-10: I61 or I63), (II) 60 years and older, (III) place of residence: Halle (Saale) and surrounding areas, (IV) adequate mobility (functional ambulation category [30] ≥ 4^1^ and self-selected walking speed > 0.4 m/s) at hospital discharge and (V) a television in the household. The exclusion criteria included (I) strong visual impairment despite visual assistance, (II) deafness or inability to communicate verbally, (III) significant mobility limitations due to comorbidity, (IV) body weight > 120 kg, epilepsy or pacemaker and (V) acute psychiatric disease or dementia.

The study nurses informed all eligible stroke patients about the study content and asked them to give their written consent for a subsequent communication. Twelve weeks after discharge from hospital, a research assistant invited all preselected persons for enrolment via a telephone. After a repeated check of inclusion criteria at the study centre, the participants provided informed consent and the baseline measurements were conducted. The waiting period between first contact (pre-selection) and the start of the study was due to the German health care system: stroke survivors usually undergo a minimum of a 3-week inpatient rehabilitation programme within the first 2 months after hospitalisation.

### Randomisation

Between the baseline data collection and the first preparation sessions at the study centre, we randomly assigned the participants into one of the two intervention arms in a 1:1 ratio. This external blinded randomisation was carried out by the Institute of Medical Epidemiology, Biostatistics, and Informatics of the Martin Luther University Halle-Wittenberg. The sport therapist informed the participants about their intervention assignment in the first preparation session.

### Trial procedure and intervention details

During the first 6 weeks, all participants received supervised balance training (according to the randomised group assignment) instructed by a sport therapist at the study centre. The training consisted of a maximum of five individual 60-min sessions with one session per week. After 6 weeks of preparation and the second data collection point (t1; 6 weeks after baseline), all participants received a one-time home visit by the sport therapist and were expected to continue the balance training independently at home for further 6 weeks. The sport therapist instructed all participants to perform their training at least three times per week for 30 min and to document all sessions in an exercise diary. During this 6-week unsupervised training phase, the sport therapist contacted all participants once a week via a phone. Furthermore, participants were covered by an accident insurance and received free tickets for public transport.

#### Arm 1: Wii™ balance intervention

For the Wii™ balance intervention, we used the Wii Fit Plus™ software (Nintendo Co. Ltd., Minami-ku Kyoto, Japan). The selection of Wii™ balance games was developed reflecting the intervention by a recent study [31]. During the preparation phase of the study (part I), the focus was on the unsupervised use of the Wii™ game console (switch on/off, software and controller use) and on testing the games ‘Ski-Slalom’, ‘Table Tilt’, ‘Penguin Slide’ and ‘Balance Bubble’ as potential intervention contents. During their home-based training (part II), the participants were able to select these balance games according to their own preference. The sport therapist installed a game console (Wii™ Mini and Wii™ Balance Board) at the one-time home visit. The game console was provided free of charge during the trial.

#### Arm 2: conventional balance intervention

The compilation of exercises for the conventional balance intervention was based on the Otago Exercise Program [32] (primarily balance, no strength exercises). It included simple balance exercises while standing and walking (e.g. weight-shifting, hip rotation, tandem/single-leg standing, tandem/backward walking, heel raises and heel/toe walking). We developed three written instructions with the same basic exercises and different levels of difficulty prior to the trial. Simple tools served to increase the difficulty of exercises (e.g. walking on a skipping rope, holding a balloon on the back of the hand during single-leg standing). All exercises were tested and trained during the preparation phase at the study centre (part 1). For their home-based training (part 2), the participants received the required material for these exercises (juggling scarves, skipping rope, small ball, balloon) and one of the three written instructions according to their physical abilities.

### Data collection and measurements

Baseline data included the socio-demographic background (e.g. age, gender, education), comorbidities (Self-Administered Comorbidity Questionnaire—German version [33]) and stroke outcomes reported by the participants (Stroke Impact Scale [34], self-reported stroke sequelae) to characterise the sample.

Feasibility of the study design was the primary concern of the pilot trial regarding (I) the target recruitment level, (II) the appropriateness of the assessments and (III) the safety, suitability and acceptance of the home-based balance training.

(I) Target recruitment level

The expected sample size was predefined in collaboration with the physicians in the study centres based on the inclusion and exclusion criteria, the hospital capacity and the expected number of patients with stroke during the recruitment period. It was estimated that about 40 stroke patients (20 each study centre) would declare their interest in participating in the trial during the 6-month recruiting period. At least 63% (*n* = 25) of the preselected and interested persons were expected to be recruited 12 weeks after discharge, and at least 80% (*n* = 20) of the study participants should complete the study in accordance with the trial protocol.

(II) Appropriateness of assessments

Assessments to investigate the efficacy of the intervention were tested at all three points in time to learn more about the instruments’ suitability for this special patient group. The selection of instruments was based on previous studies [35] including Wii™ balance training interventions. The primary efficacy outcome measurements were the Berg Balance Scale (BBS) [36], Dynamic Gait Index (DGI) [37] and the Activities-specific Balance Confidence (ABC) Scale [38]. We evaluated the appropriateness of these assessments mainly by the occurrence of ceiling effects at baseline (t0). At this time, a maximum of 20% of the study participants should achieve a value of ≥ 95% of the maximum score of the used balance assessments.

To get additional information about individual balance deficits that were not based on the expert’s rating (e.g. Berg Balance Scale and Dynamic Gait Index) or self-reported facts (e.g. ABC Scale), the Timed ‘Up and Go’ Test (TUGT) [39] and a posturographic measurement were conducted. The postural regulation was measured by the Interactive Balance System (IBS, Neurodata GmbH, Vienna, Austria). Participants stood barefoot on a force platform in eight standardised test conditions (each for 32 s). Further details regarding these measures are available elsewhere [40]. Primarily, the stability indicator (describes the postural stability state while standing) was analysed and compared to the age-adjusted values [41]. A normal stability indicator value of older adults (aged 60 or older) varies between 20 and 34 with higher values of the stability indicator pointing to a lower level of postural performance.

(III) Safety, suitability and acceptance of home-based balance training

As a criterion for feasibility of the intervention, no (self-reported) intervention-related injuries or falls should occur during the trial. Additionally, the participants were asked to evaluate the intervention via a self-constructed questionnaire after 6 weeks of unsupervised home training (t2; 12 weeks after baseline). Participants should rate their experience of stress (three items), satisfaction (four items) and perceived effects (three items) regarding the intervention, on a 5-point scale. At least 80% of participants should agree with the question: ‘It was easy to integrate the exercises into everyday life’. All participants should reach the prescribed minimum level of independent training that means 7.5 training hours (i.e. 3 × 30 min per week within at least 5 weeks). No more than 20% participants of the Wii™ balance intervention should require a second home visit due to technical problems.

### Data analyses

We used descriptive statistics to describe baseline characteristics and to summarise feasibility outcomes. To describe within-group differences between week 6 and week 12 (t1 to t2; home training phase), the change scores (95% confidence interval for mean differences) were calculated for the primary efficacy outcome measurements. Statistical analyses were conducted using SPSS for Windows, version 22.0 (SPSS Inc., Chicago, IL).

## Results

**Fig. 2:**
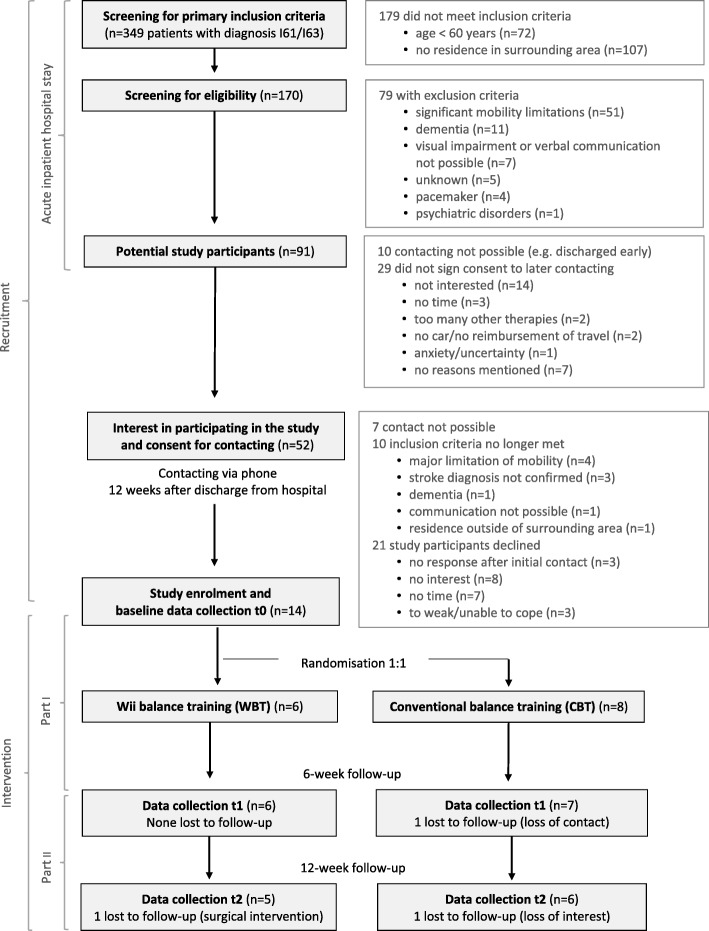
Flow chart of study participants

Within 6 months, among the 349 stroke patients screened, 91 (26%) were eligible and 52 (15% of all patients and 57% of the eligible individuals) were interested in participating in the study. After the discharge from hospital, 14 persons (4% of all persons and 27% of the eligible and interested persons) decided to participate in the study and were randomised to the Wii™ training group (*n* = 6) and the conventional balance training group (*n* = 8). Among the 52 eligible and interested patients during the recruitment in the hospital, seven persons were not available via telephone, ten no longer met inclusion criteria and 21 refused to participate. After enrolment, one participant from the Wii™ balance intervention was excluded due to a surgical intervention after t1. One participant from the conventional balance intervention was excluded from the study due to loss of contact after t0, another withdrew from the study due to lack of interest after t1 (Fig. 2).

Eleven participants completed the study (Table 1). The mean (± SD) age of participants was 74.0 (± 8.1) years, and the average time from hospitalisation to study enrolment was 18.3 (± 4.3) weeks.

**Table 1:** Sample characteristics at baseline (t0)

Participant details					Stroke details			Balance assessment				
	Age (years)	Gender	BMI (kg/m^2^)	SCQ (score)	Affected functional area (self-reported)	Time after stroke (weeks)	SIS mobility	BBS (score)	DGI (score)	ABC (score)	TUGT (s)	ST (score)
1CBT	75	m	25.9	3	UL/l	23	97	53	20	92	8.4	52.3
2CBT	72	f	26.8	10	UL + LL/r	18	97	53	23	99	6.5	32.0
3CBT	70	f	25.7	18	C + V	15	92	53	24	87	8.1	29.8
4CBT	77	m	29.4	8	LL/l	22	92	48	23	91	9.1	63.7
5CBT	63	m	31.2	4	C	23	100	56	24	99	7.7	48.5
6CBT	84	f	28.7	9	C + V	13	78	26	14	45	18.0	74.7
1WBT	85	m	28.4	2	UL/l	21	100	56	23	96	8.1	36.6
2WBT	70	m	27.8	2	UL + LL/r	19	97	55	24	97	8.6	30.9
3WBT	62	m	22.8	2	C + V	20	100	56	24	100	11.1	14.8
4WBT	71	m	26.8	2	C + V	17	100	54	23	99	7.4	30.0
5WBT	85	f	23.9	7	C + V + LL	13	56	24	5	65	(*116.0*)*	–**
Mean (SD)	74.0 (8.1)		27.0 (2.4)	6.1 (5.0)		18.6 (3.8)	91.7 (13.7)	48.5 (11.9)	20.6 (6.0)	88.2 (17.4)	9.3 (3.3)	39.0 (18.9)

Note: *CBT* conventional balance training, *WBT* Wii™ balance training, *m* male, *f* female, *UL* upper limb, *LL* lower limb, *l* left body side, *r* right body side, *C* communication, *V* vision, *SCQ* Self-Administered Comorbidity Questionnaire, *SIS* Stroke Impact Scale, *BBS* Berg Balance Scale, *DGI* Dynamic Gait Index, *ABC Scale* Activities-specific Balance Confidence Scale, *TUGT* Timed Up and Go Test, *ST* stability indicator (posturography); *the test result was not taken into account for the mean value, since the test was not carried out in accordance with the protocol at t0 (testing without walking aid although this is used continuously in everyday life); **no measurement because unsupported standing with closed eyes was not possible

The Berg Balance Scale and Dynamic Gait Index showed ceiling effects according to the definition in 73% (*n* = 8) of the participants at baseline. Regarding the ABC Scale, ceiling effects were observed in 45% (*n* = 5) of the participants. The mean (± SD) stability indicator as an indication of the motor output from postural regulation was 39.0 (± 18.9). The individual stability index exceeded the age-adjusted standard values in eight participants and indicates that the postural regulation was clearly worse in the majority of participants in comparison to normal older people.

An average of 4.4 (± 3.8) supervised training sessions were attended during the 6-week preparation phase. During home-based balance training, participants documented an average of 19.5 (± 5.9) exercise sessions with a mean total duration of 11.0 (± 6.8) hours (Table 2). Nine of 11 participants documented at least 7.5 h within the 6-week home training. Two participants of the Wii™ balance interventions documented more than 20.0 training hours. The maximum training duration documented within the conventional balance intervention was 9.3 h. In each group, one participant was below the prescribed minimum training duration (WBT 7.2 h, CBT 5.6 h).

**Table 2:** Session details for the intervention

		Total *n* = 11; mean (SD)	WBT *n* = 5; mean (SD)	CBT *n* = 6; mean (SD)
Weeks 1–6 (preparation)	Days between t0 and t1	42.5 (3.8)	43.0 (3.3)	42.0 (4.4)
	Preparation meetings, number	4.4 (0.5)	4.6 (0.5)	4.2 (0.4)
Weeks 6–12 (home-based training)	Days between t1 and t2	41.0 (4.7)	42.8 (5.5)	39.5 (3.7)
	Exercise sessions, number	19.5 (5.9)	23.4 (6.2)	16.2 (3.2)
	Exercise duration, total hours	11.0 (6.8)	14.8 (8.9)	7.8 (1.5)

Note: *WBT* Wii™ balance training, *CBT* conventional balance training, *t0* baseline, *t1* 6 weeks after baseline, *t2* 12 weeks after baseline

None of the participants reported intervention-related injuries or falls during the intervention period. One additional home visit was necessary due to operating difficulties with the television (selection of the audio-video output). Both intervention groups evaluated the home-based balance training as feasible and satisfactory (Table 3). All participants, except one, reported that independent implementation of the training programme was possible. Various trends were found in the experienced stress and perceived effects. Overall, the physical strain was rated low to moderate. On average, participants of the conventional balance intervention rated the balance requirement as rather moderate (1.7 ± 0.8) and participants of the Wii™ balance intervention as rather low (1.0 ± 0.7). Remarkably, there was less agreement in the Wii™ group on the questions of whether postural control and gait safety has improved.

**Table 3:** Participants’ evaluation of the home-based balance intervention

Dimension	Item	WBT *n* = 5; mean (SD)	CBT *n* = 6; mean (SD)
Experienced stress	Attention and concentration	2.0 (1.0)	1.8 (0.8)
	Cardiovascular stress	1.4 (0.9)	1.7 (0.8)
	Balance and coordination	1.0 (0.7)	1.7 (0.8)
Satisfaction with home-based balance training	It was fun	3.8 (0.4)	3.8 (0.4)
	It was entertaining	3.8 (0.4)	3.7 (0.5)
	It was easy to integrate into everyday life	4.0 (−)	3.8 (0.4)
	It was independently practicable	4.0 (−)	3.7 (0.8)
Perceived effects	Improved my balance sensitivity	3.0 (1.2)	3.3 (1.2)
	Improved my gait safety	2.2 (1.5)	3.3 (1.2)
	Improved my general postural control	2.4 (1.3)	3.7 (0.8)

Note: *WBT* Wii™ balance training, *CBT* conventional balance training; rating of experienced stress: (0) none, (1) low, (2) moderate, (3) high and (4) very high; rating of satisfaction and perceived effects: (0) strongly disagree, (1) disagree, (2) neither, (3) agree and (4) strongly agree

The comparisons of the feasibility aims and the results of the pilot study illustrate that the target values were not achieved for the recruitment level and the appropriateness of the assessments (Table 4). In contrast, the feasibility targets of home-based balance training were largely achieved. Both interventions seemed to be feasible among persons who completed the home-based training.

**Table 4:** Feasibility outcomes of the pilot study

Level	Parameter	Feasibility aim	Result of piloting
Target recruitment level	Interested patients in the hospital, *N*	40	52
	Recruitment rate after hospital discharge, % (*n*)	63% (25/40)	27% (14/52)
	Study completion, % (*n*)	80% (20/25)	79% (11/14)
Appropriateness of assessments	BBS Score < 53 at baseline, % (*n*)	80% (20/25)	29% (4/14)
	DGI Score < 22 at baseline, % (*n*)	80% (20/25)	29% (4/14)
	ABC Scale Score < 95 at baseline, % (*n*)	80% (20/25)	57% (8/14)
Home-based balance training	Intervention-related injuries, % (*n*)	0% (0/20)	0% (0/11)
	No problems at home^1^, % (*n*)	90% (18/20)	91% (10/11)
	At least 450 min between t1 and t2, % (*n*)	90% (18/20)	82% (9/11)
	Second home visit for technical support (only WBT), % (*n*)	20% (2/10)	20% (1/5)

Note: *BBS* Berg Balance Scale, *DGI* Dynamic Gait Index, *ABC Scale* Activities-specific Balance Confidence Scale; ^1^Definition: ‘agree’ or ‘strongly agree’ at the statement: ‘It was easy to integrate the exercises into everyday life.’; percentages for recruitment rate were based on total number of interested participants; percentages for study completion and appropriateness of assessment were based on total number of recruited participants; percentages for home-based balance training were based on total number of participants who completed the study, except home visit for technical support which was based on number of participants in the WBT intervention only

The change scores of the balance assessments between week 6 (t1) and week 12 (t2) are shown in Table 5. No reliable differences were found in the within-group analyses over time.

**Table 5:** Efficacy outcomes of the home-based training interventions at week 6 (t1) and week 12 (t2)

	Wii™ balance training			Conventional balance training		
Outcome measures	Week 6 mean (SD)	Week 12 mean (SD)	Mean difference (95% CI)	Week 6 mean (SD)	Week 12 mean (SD)	Mean difference (95% CI)
	*n* = 5	*n* = 5		*n* = 6	*n* = 6	
Berg Balance Scale	48.2 (16.3)	48.8 (15.5)	0.6 (− 1.7 to 0.5)	53.5 (3.6)	53.0 (6.4)	− 0.5 (− 3.7 to 4.7)
Dynamic Gait Index	19.8 (9.4)	20.4 (6.4)	0.6 (− 4.5 to 3.3)	21.5 (3.1)	22.0 (3.5)	0.5 (− 2.2 to 1.2)
ABC Scale	81.8 (39.4)	89.4 (23.8)	7.6 (− 27.1 to 11.8)	93.5 (12.6)	93.3 (11.5)	− 0.2 (− 1.8 to 2.3)
	*n* = 4*	*n* = 4*		*n* = 6	*n* = 6	
TUGT, s	8.4 (1.4)	7.5 (0.8)	− 0.9 (− 1.3 to 3.1)	8.2 (1.9)	8.5 (1.2)	0.3 (− 1.8 to 1.1)
Stability indicator	28.4 (9.3)	25.3 (8.9)	− 3.1 (− 2.3 to 8.5)	45.6 (19.0)	43.5 (11.4)	− 2.2 (− 7.9 to 12.3)

Note: *ABC* Activities-specific Balance Confidence, *TUGT* Timed Up and Go Test; *one WBT participant was excluded from the analysis because TUGT was measured in different settings (t1: with walker; t2: without walker) and unsupported standing with closed eyes on posturography was not possible

## Discussion

To our knowledge, this is the first pilot study that focused on the feasibility of balance training with a Nintendo Wii™ Balance Board for mobile older stroke survivors in the home setting. This involved testing the intervention in the unsupervised and less standardised condition at the participants’ homes. For comparison purposes, an intervention group with conventional balance exercises was integrated into the study design.

The main findings from this pilot study suggest that the Wii™ balance intervention seems feasible, but the tested trial procedure is not practical especially due to major problems with recruitment. The targeted stroke population could not be reached satisfactorily. We expect that the study design will not work in a multicentre context. A revision of the study design, especially concerning patient recruitment, is necessary.

Therefore, the primary value of this study arises from the findings regarding the methodological and organisational challenges in the home setting. Fifty-two out of 91 eligible stroke patients (57%) from two hospitals were interested in participating within a 6-month recruiting period. Furthermore, after 12 weeks, only 14 persons agreed to take part in the study. In the clinical setting, the rejection rates for Wii™ Balance Board studies among eligible participants varied between 14 and 31% [11, 12, 42, 43], whereas the rejection rate in our study was significantly higher (55%). A discrete choice experiment by Laver et al. [44] already indicated that the acceptance of Wii™ training in older people may be overestimated. This might explain the little interest among the possible participants in our study. Nevertheless, low recruitment levels were also reported from other home-based post-stroke studies with classical training programmes [45, 46]. A general recruitment problem might have been arisen due to the transfer of intervention into home setting. The effort for the participants to take part in this study was significantly higher than that in an inpatient setting (e.g. 12-week study duration, travelling to eight appointments in the study centre, documentation of the training sessions at home). This might have been a further reason for refusing to participate. On the other hand, once the stroke survivors had finally decided to participate in the study, they showed high adherence and completed the intervention (11 out of 14 persons).

One important exclusion criterion was the distance between the participants’ homes and the study centre for the initial preparation phase with supervision (107 of 349 persons, 31%). Therefore, future trials should find ways to include stroke survivors living within residential areas further away from the study centre. Another reason for the low recruitment rate in the study may have arisen from organisational processes. First, the declaration of interest signed by the stroke survivors in the hospital was not obligatory. Second, a long time passed between initial contact in the hospital and the start of the study in the aftercare phase. Perhaps the willingness to participate in the complex study has changed in this period due to the current health or life situation. However, the waiting period between the first contact and the start of the study was due to the German health care system: within the first month after discharge from an acute hospital, most stroke survivors in Germany spend 3 weeks in an inpatient rehabilitation centre. Indeed, this was the case for 9 of our 11 participants. Therefore, this aspect is especially relevant for home-based research in the aftercare phase following stroke in Germany. In addition, the holiday period around Christmas/New Year and individual appointment requests delayed the desired start of the intervention (planned approx. 12–14 weeks after discharge) in some cases.

Another important aspect for future intervention trials related to the functional status of the recruited sample has to be considered. Most participants had a good functional level reflected by the ceiling effects in the assessments. This leads to the question, to what extent the subjects really had clinically relevant restrictions in their balance and if they needed a balance training at all. In our home-based study, the high functional level resulted from the fact that only stroke survivors able to walk independently on hospital discharge were eligible. Therefore, the good functional level of the participants was not surprising. However, the selection of the ability to walk as an inclusion criterion was a key element in the assessment of risks and benefits for the selected study design. The participants’ safety while using the Wii™ Balance Board at home was one of the most important aspects when planning the study. The challenge of balance-related disturbances during the balance training had to be considered explicitly and led to the detailed ethical approval and the insurance cover for the participants in our home-based intervention with the Wii™ Balance Board. Considering this background, it should be noted that despite the significant ceiling effects in the established assessments, the posturographic measurement revealed balance deficits in more detail. For the majority of the participants, the results of posturography indicated a substantially worse postural regulation than in the group of healthy older people. By contrast, slight but significant impairments of balance that may affect the risk of falling in the future could not be detected with the skill-oriented standard assessments (e.g. Berg Balance Scale) used in this trial. Despite the predominantly good performance in functional tasks at present, the need for a preventive balance training seemed to be indicated using posturographic measurement. Device-based measurement methods, such as posturography or gait analysis, can generate important additional information on balance status and are likely to detect changes over time more sensitively. Due to the low level of evidence and the unclear biological mechanisms of physical adaptation through Wii™ balance training, various objective measurement methods should be integrated in future studies.

Despite the limitations in recruitment and appropriateness of the assessment, the findings of the home-based intervention are generally positive. The preceding preparation phase with a professional guide had been helpful to make the participants familiar with the technique and the exercises. This observation is consistent with the stroke-specific feasibility results in the clinical setting [11, 12, 25] and the initial results for other neurological diseases [15, 16, 18]. The participants in both intervention arms evaluated the intervention as positive, effective and feasible for self-application. No falls or injuries occurred during the intervention, whereas nearly all participants achieved the required scope of the exercises. With the exception of only one additional visit, no continued support was necessary after the introductory phase. Based on the responses of the participants, there was a tendency for the Wii™ exercise programme to be perceived as less demanding. In our intervention, the selected Wii™ games required weight-shifting movements over the Wii™ Balance Board as a fixed base. The Wii™ package offers more challenging games, but the focus was on balance, not strength or endurance exercises. Indeed, former studies reported lower levels of exertion in persons using the Wii™ compared to persons using classical forms of exercise [47–49]. This should be considered when planning comparable control conditions in intervention trials (e.g. in terms of adjusting the load). This feasibility study was not designed to prove effectiveness of the intervention. Nevertheless, information on possible intervention effects would have been desirable. On an individual level, some balance parameters showed positive trends. For example, the stability index (posturography) improved in some subjects during the study, indicating an improvement in postural control. Overall, however, the changes were inconsistent and outside the clinically relevant range. In addition to the good functional performance of the participants at baseline, the possible changes from 12 to 24 weeks after stroke might be small in this group.

Additional limitations are related to the generalizability of the study. The reached participants do not reflect the population of stroke survivors with higher functional limitations. Thus, the findings on intervention feasibility refer to a selected group of patients and should be interpreted as a first indication due to the small number of subjects. Furthermore, the duration of intervention was short and it is important to observe the acceptance and adherence over a longer period.

## Conclusion

This pilot study shows that the unsupervised use of the Wii™ Balance Board in the home environment seems possible and adequate for older stroke survivors with low functional limitations. High-quality randomised and well-planned trials are necessary to demonstrate effectiveness and to analyse additional positive effects of the Wii™ intervention compared to conventional procedures. Therefore, important study-related barriers were identified that should be considered for future study planning. Tools that assess the level of balance performance in more detail should be added to cover the functional range of study participants more effectively. Shifting recruitment and the preparation phase into rehabilitation centres or directly into the participants’ home environment seems necessary to reach more of the eligible stroke survivors.

The study shows the challenges of home-based intervention research and highlights the usefulness of pilot studies for the transition of proven inpatient interventions to the non-clinical setting. Careful methodological preparation with an analysis of feasibility parameters appears to be important, especially for a test of effectiveness under everyday conditions where high levels of standardisation cannot be granted. For future trials in this area, recommendations [50, 51] are available to improve the planning and designing of studies in the field of virtual reality and home-based interventions in rehabilitation research.

## Abbreviations

ABC Scale: Activities-specific Balance Confidence Scale
BBS: Berg Balance Scale
BMI: Body mass index
DGI: Dynamic Gait Index
SIS: Stroke Impact Scale
SQC-D: Self-Administered Comorbidity Questionnaire—German version
ST: Stability indicator (posturography outcome)
TUGT: Timed Up and Go

## Footnotes

1 The patient can walk independently on level ground, but requires help on stairs, slopes or uneven surfaces.
